# N-terminal pro-brain natriuretic peptide improves the C-ACS risk score prediction of clinical outcomes in patients with ST-elevation myocardial infarction

**DOI:** 10.1186/s12872-016-0430-0

**Published:** 2016-12-12

**Authors:** Peng-cheng He, Chong-yang Duan, Yuan-hui Liu, Xue-biao Wei, Shu-guang Lin

**Affiliations:** 1 Department of Cardiology, Guangdong Cardiovascular Institute, Guangdong Provincial Key Laboratory of Coronary Heart Disease Prevention, Guangdong General Hospital, Guangdong Academy of Medical Sciences, Guangzhou, 510080 Guangdong China; 2 State Key Laboratory of Organ Failure Research, National Clinical Research Center for Kidney Disease, Guangzhou, China; 3 Department of Biostatistics, School of Public Health, Southern Medical University, Guangzhou, 510515 China

**Keywords:** N-terminal pro-brain natriuretic peptide, Canada Acute Coronary Syndrome Risk Score, Acute ST-elevation myocardial infarction

## Abstract

**Background:**

It remained unclear whether the combination of the Canada Acute Coronary Syndrome Risk Score (CACS-RS) and N-terminal pro-brain natriuretic peptide (NT-pro-BNP) could have a better performance in predicting clinical outcomes in acute ST-elevation myocardial infarction (STEMI) patients with primary percutaneous coronary intervention.

**Methods:**

A total of 589 consecutive STEMI patients were enrolled. The potential additional predictive value of NT-pro-BNP with the CACS-RS was estimated. Primary endpoint was in-hospital mortality and long-term poor outcomes.

**Results:**

The incidence of in-hospital death was 3.1%. Patients with higher NT-pro-BNP and CACS-RS had a greater incidence of in hospital death. After adjustment for the CACS-RS, elevated NT-pro-BNP (defined as the best cutoff point based on the Youden’s index) was significantly associated with in hospital death (odd ratio = 4.55, 95%CI = 1.52–13.65, *p* = 0.007). Elevated NT-pro-BNP added to CACS-RS significantly improved the C-statistics for in-hospital death, as compared with the original score (0.762 vs. 0.683, *p* = 0.032). Furthermore, the addition of NT-pro-BNP to CACS-RS enhanced net reclassification improvement (0.901, *p* < 0.001) and integrated discrimination improvement (0.021, *p* = 0.033), suggesting effective discrimination and reclassification. In addition, the similar result was also demonstrated for in-hospital major adverse clinical events (C-statistics: 0.736 vs. 0.695, *p* = 0.017) or 3-year mortality (0.699 vs. 0.604, *p* = 0.004).

**Conclusions:**

Both NT-pro-BNP and CACS-RS are risk predictors for in hospital poor outcomes in patients with STEMI. A combination of them could derive a more accurate prediction for clinical outcome s in these patients.

## Background

Despite significant advances in treatment and prevention, patients with ST-elevation myocardial infarction (STEMI) still remained important population with high risk of adverse clinical outcomes [1], especially in developed countries [2]. Accurate and comprehensive simple risk evaluation plays an important role for these patients in appropriate therapeutic decision making. Therefore, several prognostic risk scores have been established to identify high-risk patients and provide important prognostic information, such as the Global Registry of Acute Coronary Events (GRACE) risk score [3, 4]. Recently, Fabrizio D'Ascenzo et al demonstrated that Thrombolysis in Myocardial Infarction (TIMI) and GRACE are the risk scores that up until now have been most extensively investigated, and GRACE was better than others [5]. However, these risk scores are not widely used in clinical practice because they contain many variables that may not be easily applicable before hospital admission or in the emergency department, and they require computerized calculation methods. Recently, the Canada Acute Coronary Syndrome Risk Score (CACS-RS), has been shown to permit rapid stratification of patients with acute coronary syndrome (ACS) [6]. Because this risk score is simple and easy to memorize and calculate, it can be comfortably used by health care professionals without advanced medical training. However, the predictive value of CACS-RS in selected STEMI patients remains unknown.

N-terminal-pro-brain natriuretic peptide (NT-pro-BNP) is secreted in response to cardiac hemodynamic stress mediated by volume and pressure overload [7]; NT-pro-BNP is very stable at room temperature and is often measured in clinical practices, especially in the emergency department. NT-pro-BNP has been proposed to provide prognostic information in patients with acute coronary syndrome (ACS) [8]. The current clinical cardiology guidelines also recommended the use of selected newer biomarkers, including NT-pro-BNP, to provide additional prognostic information in patients with non-ST-elevation ACS [9, 10]. However, there has been no simple and effective risk model incorporating NT-pro-BNP for predicting the prognosis of STEMI patients.

Therefore, the present study was conducted to validate the predictive value of CACS-RS for STEMI patients, and to develop a Bio-Clinical CACS-RS (Bio-C-CACS) incorporating NT-pro-BNP to evaluate whether Bio-C-CACS would improve the ability to predict clinical poor outcomes compared with CACS-RS in those patients undergoing primary percutaneous coronary intervention (PPCI).

## Methods

### Population selection

According to our institute’s protocol, we enrolled all consecutive patients who were admitted to Guangdong Cardiovascular Institute of Guangdong General Hospital, Guangdong Academy of Medical Sciences, between March 2008 and October 2012. These patients presented within 12 h of onset of cardiac symptoms with ST-segment elevation undergoing PPCI and admitted to the coronary care unit within at least 48 h of admission. Patients with cardiac shock on admission, patients with chronic peritoneal or hemodialysis treatment were excluded. Patients without pre-procedural NT-pro-BNP levels, or with severe liver or kidney dysfunction, or malignancy were also excluded.

The local ethics committee of our institute approved the study protocol. Written informed consent was obtained from the patients before the procedure, or from next of kin for patients who could not sign the informed consent themselves.

### Study protocol and Risk calculation

The baseline patient demographic data, cardiovascular risk factors, cardiac history, clinical data, and in-hospital medications of all the patients were recorded. NT-pro-BNP was measured using an electro-chemiluminescence immunoassay (Roche Diagnostics, Germany) at hospital admission before the procedure. Other clinical parameters, such as serum creatinine, cardiac troponin I, creatine kinase MB, and levels of electrolytes were measured as a part of standard clinical care. The estimated glomerular filtration rate (eGFR) was calculated using the four-variables of the Modification of Diet in Renal Disease equation for Chinese patients [11].

For each patient, we used the CACS-RS model at admission to estimate the risks for in-hospital and follow-up patient outcomes. The CACS-RS ranged from 0 to 4, with 1 point assigned for the presence of each of these variables: age ≥75 years, Killip > 1, systolic blood pressure <100 mmHg, and heart rate >100 beats/min **(**Table 1 **)**.

**Table 1:** The variables in the CACS risk score

Variables	Scores
Age ≥75 years	1
Killip > 1	1
Systolic blood pressure < 100 mmHg	1
Heart rate > 100 beats/min	1

*Abbreviation*: *CACS* Canada Acute Coronary Syndrome

### PCI procedure and medications

Primary PCI was performed with standard technique according to our institute’s protocol and AHA/ACC guidelines for the management of patients with STEMI. The use of anti-platelet agents (aspirin/clopidogrel), β-adrenergic blocking agents, angiotensin-converting enzyme inhibitors, statins, or inotropic drug support was left at the clinician’s discretion according to clinical protocols.

### Follow-up and Clinical endpoints

All patients were followed up at least 3 years after the PCI procedure. The follow up data were obtained by reviewing medical records or through a telephone interview with patients.

The primary end point was in-hospital mortality. The secondary end point was the incidence of in hospital major adverse clinical events (MACEs: including all causes mortality, nonfatal myocardial infarction, target-vessel revascularization, and cerebrovascular events) and 3-year all cause mortality [12].

### Statistical analysis

Continuous variables were expressed as mean ± standard deviation or median values with interquartile ranges (IQR), where appropriate. Categorical variables were expressed as absolute number (percentage). The Student’s *t*-test and Mann-Whitney *U* test were applied to compare normally and non-normally distributed continuous variables, respectively. The best cut-off value of NT-pro-BNP for predicting in hospital mortality was determined by the receiver-operating characteristic (ROC) curves analysis. The differences in clinical characteristics between patients with higher or lower than this cut-off value were compared. Multivariable logistic regression was performed by forward stepwise selection to evaluate the independent value of NT-pro-BNP as a categorical variable (based on the cut-off value) for in -hospital mortality, after adjusting the CACS-RS or variables, with *p* values <0.15 in the univariate analysis. Then, a new score, the Bio-C-CACS was obtained by adding the points based on the association between the CACS-RS regression coefficient and the NT-pro-BNP coefficient, if NT-pro-BNP was higher than its cut-off. The discrimination between NT-pro-BNP, CACS-RS and Bio-C-CACS risk score for in-hospital mortality or MACEs were evaluated with ROC area under the curve (AUC), sensitivity, and specificity.

The AUC was compared using the nonparametric approach of DeLong et al. [13]. Calibration was evaluated using the Hosmere-Lemeshow goodness-of-fit. We also performed net reclassification improvement (NRI) and integrated discrimination improvement (IDI) to analyze the degree to which the addition of NT-pro-BNP to the CACS-RS improved predictive ability [14]. All data analysis was performed using SAS version 9.4 (SAS Institute, Cary, NC). All statistical tests were two-tailed and statistical significance was accepted at *p* < 0.05.

## Results

### Baseline clinical characteristics and clinical outcomes

A total of 589 patients were included in the study. 16.3% were female. The percentages of patients complicated with diabetes, hypertension, and who were smokers were 21.6%, 54.3% and 48.9%, respectively. The mean age was 63.0 ± 11.9 years, mean eGFR was 77.70 ± 26.5 mL/min/1.73m^2^. NT-pro-BNP showed a median of 1244 pg/mL (IQR = 515-2704). The CACS-RS showed a median of 1 (IQR = 0-1), with 45.84% being low risk (0-1), 51.61% medium risk (1-3) and 2.55% high risk (≥3).

From the CACS-RS low risk to high risk, there was a positive trend with older age, NT-pro-BNP levels, and the pre-procedural SCr level. There was a negative trend with the pre-procedural renal function and left ventricular ejection fraction (LVEF). However, there were no significant differences in the incidence of hypertension, diabetes, or previous myocardial infarction among the different risk groups of CACS- RS (Table 2).

**Table 2:** Baseline characteristics of patients according to C-ACS-RS group

Variables	0 (*n* = 266)	1 (*n* = 217)	2 (*n* = 95)	≥3 (*n* = 11)	*P* value
Demographics					
Age, years	58.32 ± 9.84	64.41 ± 11.93	71.08 ± 11.22	77.55 ± 6.31	<0.001
Age ≥ 75 years, n (%)	0 (0.0%)	46 (21.2%)	45 (47.4%)	8 (72.7%)	<0.001
Female, n (%)	34 (12.8%)	41 (18.9%)	20 (21.1%)	1 (9.1%)	0.140
Systolic BP (mmHg)	125.36 ± 16.16	117.79 ± 21.45	113.28 ± 29.40	98.30 ± 27.75	<0.001
Diastolic BP (mmHg)	76.22 ± 40.09	71.02 ± 14.00	67.00 ± 16.34	59.70 ± 14.49	0.028
Heart rate (beat/min)	76.88 ± 11.23	79.77 ± 16.92	85.70 ± 24.54	86.70 ± 18.56	<0.001
Medical history, n (%)					
Diabetes	56 (21.1%)	48 (22.1%)	19 (20.0%)	4 (36.4%)	0.760
Previous myocardial infarction	12 (4.5%)	14 (6.5%)	5 (5.3%)	2 (18.2%)	0.240
Coronary artery bypass graft	11 (4.1%)	6 (2.8%)	10(10.5%)	2(18.2%)	0.005
Hypertension	135 (50.8%)	121 (55.8%)	56 (58.9%)	8 (72.7%)	0.276
Smoking	138 (51.9%)	102 (47.0%)	43 (45.3%)	5 (45.5%)	0.612
Anemia	78 (29.3%)	45 (20.7%)	16 (16.8%)	2 (18.2%)	0.040
Laboratory findings					
NT-pro-BNP, pg/mL(Median)	851.15	1506.00	2414.00	2330.00	<0.001
Lg NT-pro-BNP, pg/mL	6.61 ± 1.27	7.28 ± 1.23	7.81 ± 1.26	7.85 ± 1.46	<0.001
Pre-procedural SCr (μmol/L)	91.24 ± 44.05	101.04 ± 39.58	111.49 ± 52.16	171.92 ± 147.90	<0.001
eGFR, mL/min/1.73 m^2^	90.59 ± 94.56	78.16 ± 38.82	66.34 ± 21.50	50.97 ± 26.01	0.008
LVEF, %	55.54 ± 10.42	53.54 ± 10.31	49.04 ± 10.85	51.67 ± 14.62	<0.001
Hemoglobin (g/L)	135.33 ± 16.32	131.13 ± 17.97	127.39 ± 17.10	132.52 ± 18.79	<0.001
Hemoglobin A1c (%)	6.47 ± 1.36	6.62 ± 1.74	6.30 ± 1.39	6.44 ± 0.64	0.554
Serum albumin (g/L)	34.47 ± 5.24	32.71 ± 4.30	31.58 ± 4.47	31.27 ± 4.85	<0.001
Uric acid (μmol/L)	358.13 ± 93.3	377.69 ± 124.2	380.06 ± 117.7	393.11 ± 79.9	0.265
Procedural characteristic					
Contrast volume (mL)	132.80 ± 53.81	132.92 ± 53.00	144.29 ± 43.39	187.50 ± 81.32	0.291
Contrast exposure time (min)	78.89 ± 42.27	82.52 ± 37.72	92.02 ± 42.80	80.00 ± 49.50	0.347
Number of diseased vessels (n)	1.99 ± 1.17	2.08 ± 0.90	2.26 ± 0.94	2.00 ± 0.77	0.197
Number of stents (n)	1.36 ± 0.82	1.40 ± 0.77	1.49 ± 0.84	1.36 ± 0.50	0.587
Total length of stent (mm)	37.04 ± 26.47	35.61 ± 23.55	37.05 ± 24.60	24.00 ± 8.49	0.871

*Abbreviation*: *C-ACS-RS* Canada Acute Coronary Syndrome risk score, *NT-pro-BNP* N-terminal-pro-brain natriuretic peptide, *SCr* serum creatinine, *eGFR* estimated glomerular filtration rate, *LVEF* left ventricular ejected function

Overall, the incidence of in-hospital mortality was 3.1%, and the MACEs were 23.8%. The median follow-up period was 3.54 ± 1.40 years (inter quartile range, 2.61–4.28 years). During patient follow up, 3-year all cause mortality developed in 26 patients (5.9%).

### Predictive value of CACS-RS

**Fig. 1:**
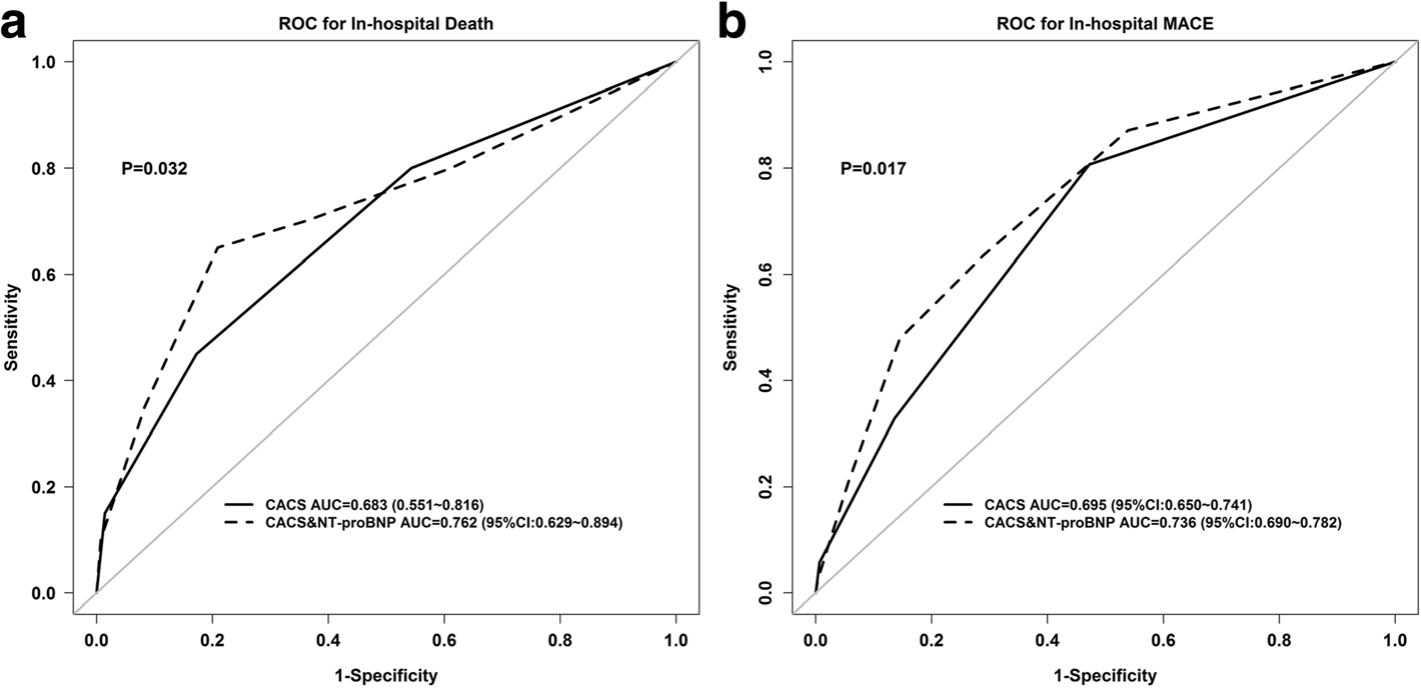
Area under the receiver operating characteristic curve of the C-ACS and Bio-C-CACS-RS group for predicting in-hospital mortality (**a**) and major clinical adverse events (**b**)

Patients who developed in-hospital mortality presented with a higher CACS-RS than those without (1.50 vs. 0.71, *p* = 0.008). The similar results were also demonstrated in patients developed in hospital MACEs or 3-year mortality (1.21 vs. 0.59, *p* < 0.001; 1.16 vs. 0.67, *p* < 0.001). The predictive value of CACS-RS for in hospital mortality was 0.683 (95% CI = 0.551-0.816) (Fig. 1 **)**. CACS-RS also showed predictive accuracy for in hospital MACEs **(**Fig. 1 **)** or 3-year all cause mortality, with C-statistics of 0.695 (95% CI = 0.650-0.741), 0.604(95% CI = 0.515- 0.694).

### Independent Predictive value of NT-pro-BNP

In addition, the best cut-off value of NT-pro-BNP for predicting in-hospital mortality was 2300 pg/mL with 72.2% sensitivity and 73.0% specificity, based on the Youden index. Furthermore, comparing to patients with low NT-pro-BNP (<2300 pg/mL), patients with NT-pro-BNP ≥2300 pg/mL presented with a significantly higher in-hospital mortality (7.74% vs. 1.19%, *p* < 0.001) or in hospital MACEs (42.86% vs. 16.15%, *p* < 0.001). The Kaplan-Meier curve showed that the incidence of MACEs was higher in those patients with higher NT-pro-BNP levels. Log-rank test on the curves demonstrated significant difference between two groups (Chi square = 15.56, *P* < 0.001).

Univariate logistic regression analysis showed that NT-pro-BNP ≥2300 pg/mL was significantly associated with in-hospital mortality (OR = 6.98, 95% CI = 2.45–19.90, *p* < 0.001). Additional significant variables included CACS-RS (OR = 2.76, 95% CI, 1.64–4.66, *p* < 0.001). The multivariate analysis, together with CACS-RS and NT-pro-BNP (as a categorical variable) demonstrated that CACS-RS and NT-pro- BNP ≥2300 pg/mL remained the significant independent predictor of in hospital mortality (OR = 2.15, 95%CI, 1.24–3.75, *p* = 0.007; OR = 4.55, 95% CI, 1.52–13.65, *p* = 0.007).

### Combination of NT-pro-BNP with the CACS-RS

**Fig. 2:**
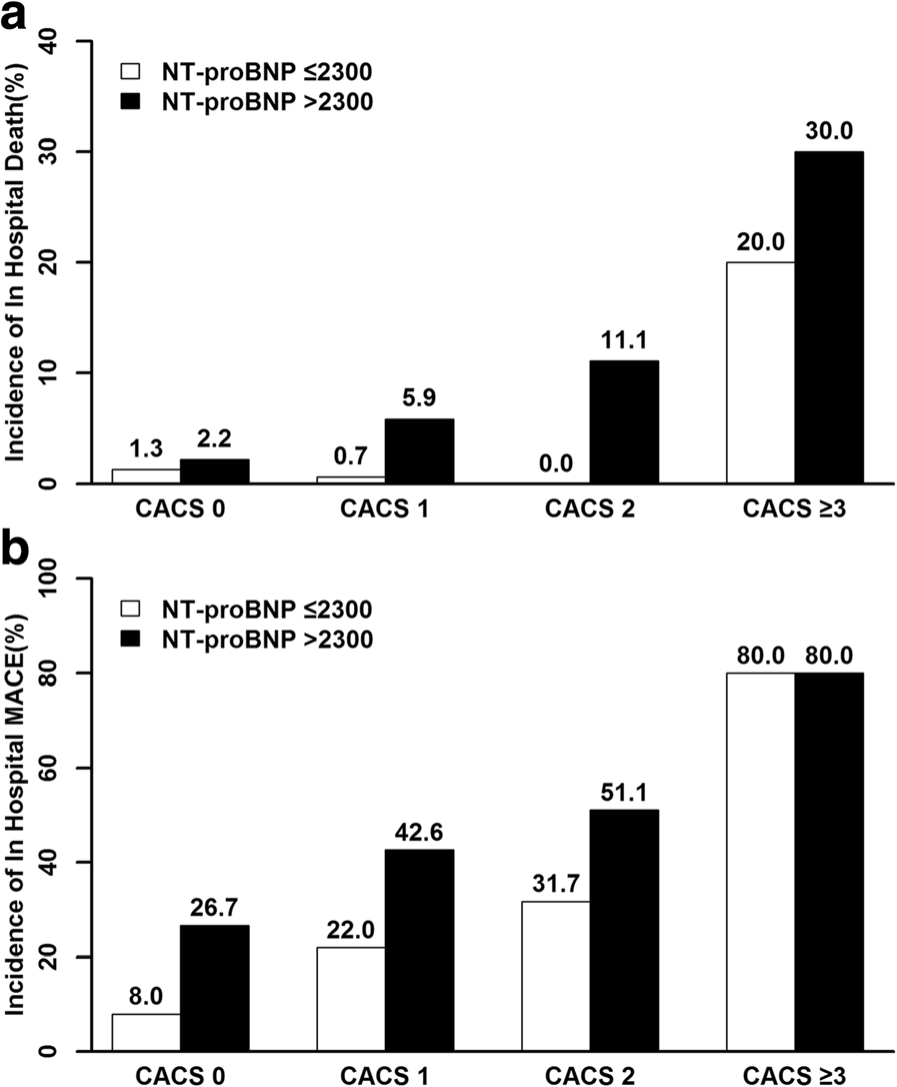
Incidence of in-hospital mortality (**a**) and major clinical adverse events (**b**) according to different C-ACS-RS group or Bio-C-CACS-RS group

In order to evaluate the additional predictive value of NT-pro-BNP to CACS-RS, the NT-pro-BNP (as a categorical variable, according to the cut-off value) was incorporated into the new score (Bio-C-CACS-RS). Combinations of NT-pro-BNP with CACS-RS might more accurately identify patients at high risk of in hospital mortality or MACEs than using CACS-RS only. (Fig. 2 **)**

In addition, ROC analysis demonstrated that the AUC for in hospital mortality increased significantly after the addition of NT-pro-BNP to the CACS-RS (AUC: 0.762 vs. 0.683; *p* = 0.032), as did the Hosmer-Lemeshow goodness of fit (*X* ^2^ = 7.44, *p* = 0.489). (Fig. 1 **)** More importantly, the inclusion of NT-pro-BNP into the CACS-RS was associated with a NRI of 90.1%, suggesting effective reclassification. The IDI showed that the model diagnostic performance was significantly improved by adding NT-pro-BNP to the CACS-RS (IDI = 0.021, *p* = 0.033).

Meanwhile, applying the same statistic metrics to other clinical endpoints, we found that NT-pro-BNP increased the AUC, and improved the reclassification and discrimination ability when added to the CACS-RS, with in-hospital MACEs: (AUC: 0.736 vs. 0.695, IDI: 0.032, NRI: 0.601); 3-year all cause mortality: (AUC: 0.699 vs. 0.604, IDI: 0.032, NRI: 0.762).

## Discussions

This study demonstrated that CACS-RS is an independent predictor of outcomes in STEMI patients undergoing PPCI, and with good predictive value of poor outcomes. Furthermore, this might be the first study to demonstrate that the measurement of NT-pro-BNP concentrations on patient hospital admission add prognostic information about short- and long-term outcomes to the CACS-RS. This study has described the use of the new Bio-C-CACS.

STEMI patients remain an important clinical population with a risk of adverse clinical outcomes [2]. In the present study, the in hospital mortality of STEMI patients was shown to be 3.1% and the 3-year mortality was 5.9%, which was lower than the incidence of mortality in the study by Campo G et al [15–17]. It might be related to the different percentage of hypertension, previous myocardial infraction and the type of stent. The findings in present and previous studies support the aim of this study, to develop improved clinical tools to identify STEMI patients at high risk of poor clinical outcome. Accurate and comprehensive simple risk evaluation plays an important role for these patients in appropriate therapeutic decision making. Higher risk scores usually imply that higher-intensity treatments may be appropriate within the context of the patient’s health status. However, inappropriate use of aggressive medical management in patients at low-risks may only expose them to experience adverse effects.

Several risk-scoring systems have been proven to evaluate the risk of poor clinical outcomes in STEMI patients. The GRACE risk score is one of the most frequently used models, incorporating clinical investigation (such as an ECG) and cardiac and renal biomarker (such as creatinine kinase MB and serum creatinine levels). However, the GRACE risk score requires computerized calculation methods, and not all clinical information for this assessment may be available at first clinical contact. In addition, the TIMI score for STEMI is an another popular risk-assessment tool, which is simpler to use than the GRACE score, but may also require the availability of an ECG and patient weight on admission [18]. Furthermore, previous research has shown that the Mehran risk score (MRS) for contrast-induced nephropathy can be applied to stratify STEMI patients for poor clinical outcomes both in the short- and long-term follow-up. However, the MRS incorporates eight variables, which include not only the history of previous diseases, but also the procedure-related variables (such as contrast volume), and cannot be used before the procedure [19]. The clinical SYNTAX risk score is used for identifying STEMI patients for poor clinical outcomes, and was based on the anatomy of the coronary diseases following coronary angiography, but this scoring method cannot be used in clinical practice before the PCI.

Although the above risk-scoring systems were demonstrated the good predictive value for the clinical outcomes for STEMI patients, they are limited due to their relative complexity, the requirement of data calculation, and the required the procedure related variables. In contrast, the CACS-RS only requires basic demographic and initial hemodynamic data, which can be acquired in the emergency department, or possibly prior to arrival at the hospital.

Despite its simplicity, the CACS-RS had good predictive value for clinical outcomes. The C statistic of in hospital mortality was 0.683. The CACS-RS was first developed by Huynh et al, who performed their research study to include the ACS patients, most of whom were without ST-segment elevation; the score was demonstrated to have good predictive values for short- and long-term mortality of ACS patients [6]. The C statistic in this previous study was similar to the findings in the present study (0.73 vs. 0.68), which included only STEMI patients. More recently, two published studies have validated the clinical usefulness of CACS-RS in ACS patients. One study reported that CACS-RS performed well in predicting hospital mortality in a contemporary ACS population outside North America [20]. The other study showed that CACS-RS was the strongest predictor of in-hospital mortality in all ACS patients in western Romania [21]. However, we propose that the present study is the first to further validate the predictive value of the C-ACS score in a selected STEMI patient population. The difference in C-statistic analysis among these researches might be related to the differences in patient populations studied, and on the characters of the patients included in the studies. However, the CACS-RS had acceptable predictive value for STEMI patients, and permits rapid stratification of patients with STEMI, and would be welcomed for used by busy clinicians, because it is simple and can be used as an initial risk-assessment tools by health care professionals without advanced medical training.

In addition, although more biomarkers are being added to develop risk clinical scoring systems, many new biomarkers still have not been taken account into the CACS-RS. NT-pro-BNP, which is influenced both by cardiac and renal function, can be quickly measured by the bedside, and is increasingly shown to be predictive of short- and long-term outcomes following STEMI [22]. The current clinical guidelines also recommended that the use of selected newer biomarkers, especially NT-pro-BNP, may provide additional prognostic information in patients with non–ST-elevation ACS. Lee et al found that an improvement in the ability of the clinical SYNTAX score to predict 1-year major adverse cardiovascular events can be achieved by combining the clinical SYNTAX score with an NT-pro-BNP [23]. Similar results have been found in the study performed by Grabowski et al. Admission of BNP adds significant prognostic information in addition to that of Killip classes and TIMI risk score in STEMI patients [24]. However, another study showed that NT-pro-BNP did not increase the prognostic accuracy of the GRACE risk score in patients with ACS [25]. To date, it has been unclear whether NT-pro-BNP could provide additional predictive value for CACS-RS. The present study found that adding the NT-pro-BNP to the CACS-RS could increase the predictive value for patient clinical outcome. This is unsurprising, because STEMI patients with significant left ventricular dysfunction appear to be at low risk based on the CACS-RS if the blood pressure or heart rate is within the normal range, but the risk increase with increased NT-pro-BNP levels.

It is important to bear in mind that risk scores only based on the clinical characteristic are supplementary tools and are no replacement for clinical judgment or biomarker measurement, but combining them could have a beneficial cumulative effect. According to the guideline’s recommendation that risk assessment is a continuous process that should be repeated throughout the hospitalization duration and at time of discharge, after we easily used the CACS-RS to identify patients at risk of poor clinical outcome at the first medical contact, we should re-calculate the CACS-RS, and add the NT-pro-BNP to the CACS-RS to evaluate the risks for patients during in-hospital stay or following hospital discharge.

### Clinical implications

The results of the present study may have important clinical implications. The C-ACS-RS permits rapid stratification of STEMI patients. Because it is simple and easy to memorize and calculate, it can be rapidly applied at the first medical contact. In particular, the combined application of the C-ACSRS with the plasma NT-pro-BNP levels on admission serves to identify high-risk patients. The effective risk stratification provided may be of specific value for early therapeutic decision making and patient treatment in the different risk of STEMI patients.

### Limitations

The current study had several limitations. Firstly, It was a single-center, observational study, including a relatively small number of STEMI patients. The results of a single study should be interpreted with caution. In addition, we did not measure NT-pro-BNP concentrations at long-term follow up, such as at 3 months or at 1 year. Thirdly, C-ACS-Rs lacks precision, being more of a categorical than a continuous scoring system. The Killip class evaluation is totally dependent on the clinical evaluation and expertise of the examiner. However, this scoring system is simple and easy to apply.

## Conclusions

In conclusion, for the fist time, the present study validated the predictive value of C-ACS-RS in STEMI patients. The combination of C-ACS-RS and NT-pro-BNP could result in a more accurate prediction for clinical outcomes in these patients.
